# The complete chloroplast genome sequence of *Camellia sinensis* cultivar ‘Qiancha1’ from Guizhou Province, China

**DOI:** 10.1080/23802359.2021.2005490

**Published:** 2022-02-20

**Authors:** Chun Yang, Dahe Qiao, Yan Guo, Juan Chen, Zhengwu Chen

**Affiliations:** Tea Research Institute, Guizhou Academy of Agricultural Sciences, Guiyang, China

**Keywords:** *Camellia sinensis*, chloroplast genome, Qiancha1

## Abstract

The complete chloroplast genome sequence of *Camellia sinensis* cultivar ‘Qiancha 1’ (QC1), an excellent tea plant cultivar was determined in this study. The cp genome of QC1 is 157,024 bp in length and includes a large single copy (LSC, 86,585 bp), a small single copy (SSC, 18,277 bp) and a pair of inverted repeats (IRa and IRb, 26,081 bp). The overall GC content is 37.3%. A total of 137 genes were annotated, including 92 protein coding, 37 tRNA, and eight rRNA genes. Phylogenetic analysis showed that QC1 has the closest evolutionary relationship with *C. sinensis* cultivar ‘Anhua’ from Hunan, China. The complete cp genome of QC1 provides a resource for further research on the phylogeny and taxonomy of Sect. *Thea* (L.) Dyer.

*Camellia sinensis* cultivar ‘Qiancha 1’ (QC1) is a shrub type clonal tea plant [*Camellia sinensis* (L.) O. Kuntze] selected from ‘Meitantaicha’ (a local species of Guizhou), which has been registered by the Ministry of Agriculture and Rural Affairs of the People’s Republic of China (registration number: GPD Tea plant (2019) 520007, http://202.127.42.47:6010/index.aspx) (Yang et al. 2019). The cultivar QC1 is suitable for making premium green tea as well as for the production of high-quality black and white teas (Li et al. 2018). In addition, QC1 also has a high transplanting survival rate, strong growth potential and high yield, and is welcomed by tea growers and consumers (Yang et al. 2019). At present, QC1 is being used as a parent for cross breeding with other cultivars, but its genetic background has not been fully documented. Considering the accuracy and necessity of the chloroplast (cp) genome in identifying the origin of parents from different cultivars, the complete cp genome of QC1 was sequenced and analyzed in this study.

The young leaves of QC1 were collected from a nine-year old individual, growing in the germplasm tea repository of Guizhou tea research institute located at Guiyang (N26°30′, E106°39′), Guizhou Province, China. A voucher specimen (GZCS0023) was deposited in the Laboratory of Guizhou Tea Germplasm Innovation Engineering Technology Research Center of Tea Research Institute of the Guizhou Academy of Agricultural Sciences (http://www.gznkycys.cn/, Zhengwu Chen, zwchentea@163.com). The total genomic DNA of QC1 was isolated using the Plant Genomic DNA Kit (TIANGEN, Beijing, China) according to the manufacturer’s instructions, and sequencing was performed on the Illumina Novaseq6000 platform based on the Paired-End 150 (PE150) strategy. The cp genome was *de novo* assembled using the default settings in SPAdes v.3.5.0 (Lapidus et al. 2014) and annotated using CpGAVAS2 (Shi et al. 2019) and ORF Finder. The blastn and blastp methods were used to compare the preliminary annotated results with the reported proteins and rRNAs of cp genomes of related species to verify the accuracy of the annotation.

The complete chloroplast genome of QC1 is 157,024 bp in length and includes a large single copy (LSC, 86,585 bp), a small single copy (SSC, 18,277 bp) and a pair of inverted repeats (IRa and IRb, 26,081 bp). The GC content of the LSC, SSC, and two IRs are 35.3%, 30.6%, and 43.0%, respectively, while the overall GC content of the complete chloroplast genome is 37.3%. A total of 137 genes were annotated, including 92 protein-coding, 37 tRNA, and eight rRNA genes. Among them, 21 genes (14 protein-coding and seven tRNA) contained introns. All but two genes contained one intron, *ycf3* and *clpP* displayed two introns. The *ycf2* gene located in the IR region had the longest sequence and encoded a protein of 2298 amino acids. The complete annotated chloroplast genome sequence of QC1 was deposited to NCBI GenBank with the accession number MZ043860.

To confirm the phylogenetic position of QC1, the complete chloroplast genome sequences of QC1 and 13 published chloroplast genome sequences from Sect. *Thea* (L.) Dyer was aligned using the auto setting in MAFFT v7.475 (Nakamura et al. 2018). The complete chloroplast genome of *Ficus formosana* was designated as outgroup and a phylogenetic tree was reconstructed based on the Neighbour-joining (NJ) method with 1000 bootstrap replicates in MEGA6 (Tamura et al. 2013). The result showed that the two main cultivated types (CSS and CSA) of tea plants (Wei et al. 2018) are distinguished from the wild type and that QC1 was fully resolved in a clade with *C. sinensis* var. sinensis cv. ‘Anhua’ (Figure 1). Although there are no breeding records showing that these two varieties from different provinces share the same parental origin, our previous studies based on genomic SNPs suggest that QC1 is closely genetically related to Fudingdabaicha (Qiao et al. 2019). Since Fudingdabaicha is one of the most widely used parents in Chinese tea breeding (Zhang et al. 2020), we speculate that QC1 and 'Anhua' may be its progeny, which needs to be further confirmed by the chloroplast genome of Fudingdabaicha. The complete cp genome of QC1 also provides a resource for further research on the phylogeny and taxonomy of Sect. *Thea* (L.) Dyer plants.

**Figure 1.**
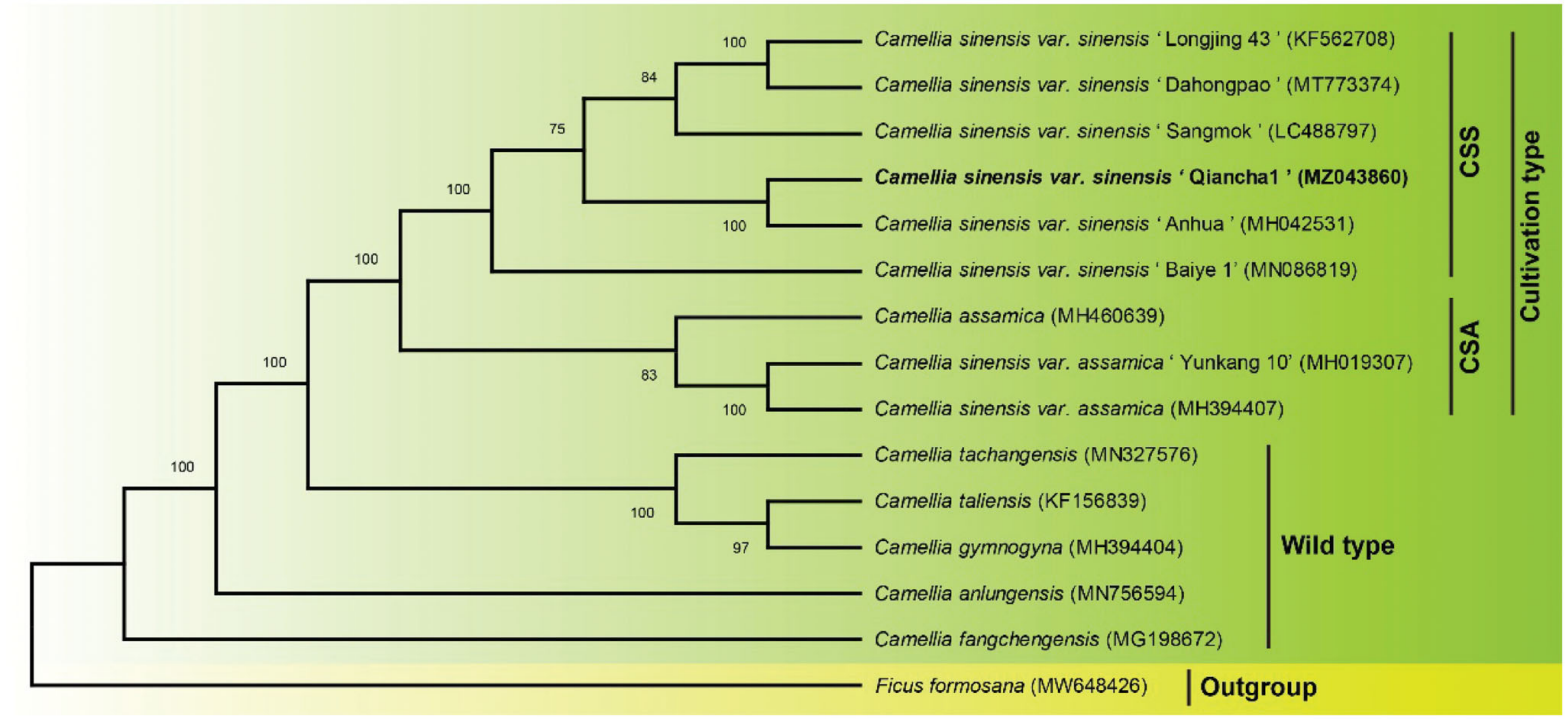
Phylogenetic relationships of 14 Sect. *Thea* (L.) Dyer resources based on their complete chloroplast genome sequences. CSS: *C. sinensis* var. *sinensis*; CSA: *C. sinensis* var. *assamica*.

## Data Availability

The data that support the findings of this study are openly available in GenBank of NCBI at https://www.ncbi.nlm.nih.gov/, under the accession no. MZ043860. The associated BioProject, SRA, and Bio-Sample numbers are PRJNA735884, SRR14756203, and SAMN19600302, respectively. The data that newly obtained at this study are also publicly available in the National Genomics Data Center at https://ngdc.cncb.ac.cn under the accession number of GWHBECZ00000000.
